# Examining the benefits of industry clinical placements for medical laboratory science academics

**DOI:** 10.1093/labmed/lmag027

**Published:** 2026-05-10

**Authors:** Dodie Pouniotis, Lynda Sharp, Ross Vlahos, Sapha Shibeeb

**Affiliations:** School of Health and Biomedical Sciences, RMIT University, Bundoora, VIC, Australia; Discipline of Laboratory Medicine, RMIT University, Bundoora, VIC, Australia; School of Health and Biomedical Sciences, RMIT University, Bundoora, VIC, Australia; School of Health and Biomedical Sciences, RMIT University, Bundoora, VIC, Australia; Centre for Respiratory Science & Health, RMIT University, Bundoora, VIC, Australia; School of Health and Biomedical Sciences, RMIT University, Bundoora, VIC, Australia; Discipline of Laboratory Medicine, RMIT University, Bundoora, VIC, Australia

**Keywords:** medical laboratory science education, work-integrated learning, academic development, university-industry partnerships

## Abstract

**Introduction:**

Industry placements are central to Medical Laboratory Science (MLS) student training, yet their value as professional development for MLS academics has not been well described.

**Methods:**

A cross-sectional survey was conducted across Australian universities offering Australian Institute of Medical and Clinical Scientists–accredited MLS programs. Two online surveys captured MLS academics’ and program leaders’ perceptions of the benefits, feasibility, and implementation considerations of academic clinical placements.

**Results:**

Among 29 academics (35% response rate) and 10 program leaders (83% response rate), hematology was the most common teaching area (30%). Most (79%) academics reported prior clinical pathology experience, with more than half reporting at least 10 years of experience. Maintaining clinical currency was rated extremely important, and placements were perceived as beneficial for teaching and student learning. Preferences centered on short placements (≤2 weeks), conducted annually or biannually, and participation likelihood increased with departmental support and short durations. Program leaders expressed unanimous support for placements to enhance curriculum alignment, student engagement, and technological currency. They also recognized the benefit for university-industry partnerships and skill development.

**Discussion:**

Academic clinical placements are widely perceived by both academics and program leaders as educationally valuable, enhancing teaching, student learning, and curriculum alignment with industry practices.

## Introduction

Medical laboratory scientists are central to health care, performing diagnostic tests that inform up to 70% of clinical decisions.^1^ Medical Laboratory Science (MLS) and Laboratory Medicine programs in Australia are structured to include substantial clinical placement or work-integrated learning, ensuring that graduates develop practical skills, professional behaviors, and work readiness in addition to theoretical knowledge.^2^^,^^3^ Student clinical placements are widely recognized for consolidating discipline-specific skills, understanding workflows and quality systems, and facilitating interprofessional engagement in real-world environments.^4^ Accreditation requirements specify minimum placement durations and competencies, illustrating sectorwide consensus on the educational value of authentic workplace learning for MLS students. Research across health professions further highlights that well-designed work-integrated learning improves employability, professional identity formation, and smooth transition into practice.^5^^,^^6^

Although the benefits of clinical placements for students are well documented, the value of such placements for professional development of MLS academics has been less well explored. There is increasing emphasis on aligning academic work and professional development with industry needs through structured engagement, partnerships, and co-designed curricula in health education.^7^ Industry engagement initiatives deliver curriculum enrichment,^8^ improved academic currency and engagement, expanded resource access, enhanced networking, and increased teaching effectiveness, potentially translating to improved student outcomes.^8^^,^^9^

In MLS, rapid technological and organizational changes, such as advances in automation, digital pathology, and quality assurance frameworks, drive continual updates to curricula and teaching practices.^10^ Many MLS academics enter academics with clinical backgrounds, but teaching, research, and administrative demands can restrict ongoing engagement with current laboratory practice.^11^ Short-term industry placements offer opportunities for academics to reconnect with contemporary workflows, technologies, and culture, strengthening professional development and enhancing the authenticity of student learning.^12^^,^^13^ Placement experiences enable academics to observe analytical workflows, accreditation practices, and interprofessional communication, informing more authentic teaching materials and curriculum development.^11^^,^^13^ Such engagement fosters collaborative curriculum review and mutual understanding between universities and industry partners, aligning learning outcomes with contemporary standards and workplace needs.

The literature emphasizes reciprocal benefits for educators and industry, including shared responsibility for graduate preparedness and opportunities for co-developed innovation.^14^ Medical Laboratory Science educators see themselves not only as technical teachers but also as mentors who transmit tacit professional knowledge and norms.^15^ Extending this perspective, industry placements may help academics develop integrated teacher-practitioner identities and reinforce their role within a broader community of practice. Despite this trend, little empirical evidence exists on MLS academics’ perceptions of undertaking clinical placements for professional development.^11^ Most published MLS education research focuses on student perspectives and career paths,^16–18^ with no systematic assessment of the benefits, challenges, and outcomes of industry placement experiences for academics.

Australian MLS programs are accredited by the Australian Institute of Medical and Clinical Scientists (AIMS), which enforces standards to ensure graduate readiness for current practice.^11^ Although student placements are mandated, there is no formal framework for academic staff engagement with industry. Maintaining clinical currency remains an individual responsibility amid teaching academics, despite increasing demands related to teaching, research, and service. This context highlights the importance of exploring structured industry clinical placements as a potential professional development strategy for MLS academics within AIMS-accredited programs. This study aimed to investigate MLS academics’ and program leaders’ views regarding industry clinical placements for academics, focusing on perceived benefits for professional development, impacts on teaching effectiveness, and implementation challenges in the context of AIMS-accredited programs.

## Methods

### Study design

A cross-sectional survey was conducted to examine perceptions of industry clinical placements for MLS academics and program leaders. In this study, *industry* refers to diagnostic pathology laboratories, including public hospital and private pathology laboratories (and, where applicable, reference laboratories) commonly involved in MLS clinical training. Forensic laboratories and in vitro diagnostics and manufacturing settings were not the focus of this study. Here, *MLS academics* refers to university-employed teaching staff who teach into AIMS-accredited MLS programs and does not include laboratory-employed clinical preceptors or instructors.

Two complementary online surveys captured perspectives at both the individual academic and program leadership levels. The study was conducted across Australian universities offering AIMS-accredited MLS undergraduate and postgraduate programs.

### Participant recruitment and data collection

Participants were drawn from Australian institutions that deliver AIMS-accredited MLS programs. The study consisted of 2 groups:

- MLS academics teaching in AIMS-accredited undergraduate and/or postgraduate programs
- Program leaders (eg, program directors, discipline leads, or heads of school) overseeing AIMS-accredited MLS programs

Surveys were designed and distributed through Qualtrics Survey using institutional email lists. Participation was voluntary, with implied consent provided through survey completion. Reminder emails were issued to maximize response rates. A total of 83 MLS academics and 12 program leaders were invited to participate. Completed surveys were received from 29 (35%) academics and 10 (83%) program leaders.

### Survey instruments

Two purpose-designed online questionnaires were developed based on literature regarding academic industry engagement and professional development. Survey items framed academic placements as a voluntary professional development opportunity and assessed interest, willingness, and likelihood of participation under different feasibility conditions (eg, duration and departmental support).

Both surveys consisted primarily of multiple-choice items and 5-point Likert-scale questions, with opportunities for optional qualitative comments. The academic survey collected information about demographics, prior clinical laboratory experience, current engagement with industry, perceptions of the benefits of clinical placements (professional development and teaching effectiveness), interest in participating, and perceived barriers to implementation. The program leader survey addressed perceptions of the value of academic clinical placements for curriculum relevance, academic capability, university-industry partnerships, institutional and industry capacity, and overall support for implementation. Most questions were multiple-choice or 5-point Likert-scale, with space for optional qualitative comments.

### Ethics

Ethics approval was obtained from the institutional Human Research Ethics Committee (No. 2025-28145-27288). All responses were anonymous, and no identifying information was collected.

### Statistical analysis

Quantitative data were analyzed descriptively. Frequencies and percentages were calculated for categorical and Likert-scale responses. Due to the exploratory nature and sample size of the study, inferential statistical analyses were not conducted. Survey responses were analyzed separately, then compared thematically to identify areas of convergence and divergence between academics and program leaders.

## Results

### Participant characteristics

Invitations were distributed across multiple Australian universities offering AIMS-accredited MLS programs. Institution identifiers were not collected to preserve anonymity; therefore, institution-level response distribution cannot be reported. A total of 29 MLS academics participated in the survey, representing a response rate of 35% (29/83 invited). **Table 1** summarizes participants’ demographic and professional backgrounds. The most commonly reported primary teaching area was hematology (9 respondents [30%]), while the remaining 70% (20 respondents) taught in other MLS disciplines. Prior clinical pathology experience was common, with 23 (79%) academics indicating such a background; 6 (21%) academics reported no prior clinical experience. Among respondents with clinical experience, 8 (35%) had 1 to 5 years, 2 (9%) had 5 to 10 years, 8 (35%) had more than 10 years, and 5 (21%) had more than 20 years of experience. A small number of respondents (2/29 [7%]) reported concurrent employment in a medical laboratory in addition to their academic role. Given the small number, subgroup comparisons were not performed.

**Table 1:** Demographic and professional background of MLS academics respondents.

Attribute	Response, No. (%)
Primary teaching area	
Hematology	9 (31)
Other MLS disciplines	20 (69)
Experience in clinical pathology	
Yes	23 (79)
No	6 (21)
Duration of clinical experience (for individuals with experience), y	
1-5	8 (35)
6-10	2 (9)
11-19	8 (35)
>20	5 (21)

Abbreviation: MLS, Medical Laboratory Science.

### Industry currency and engagement

**Figure 1:**
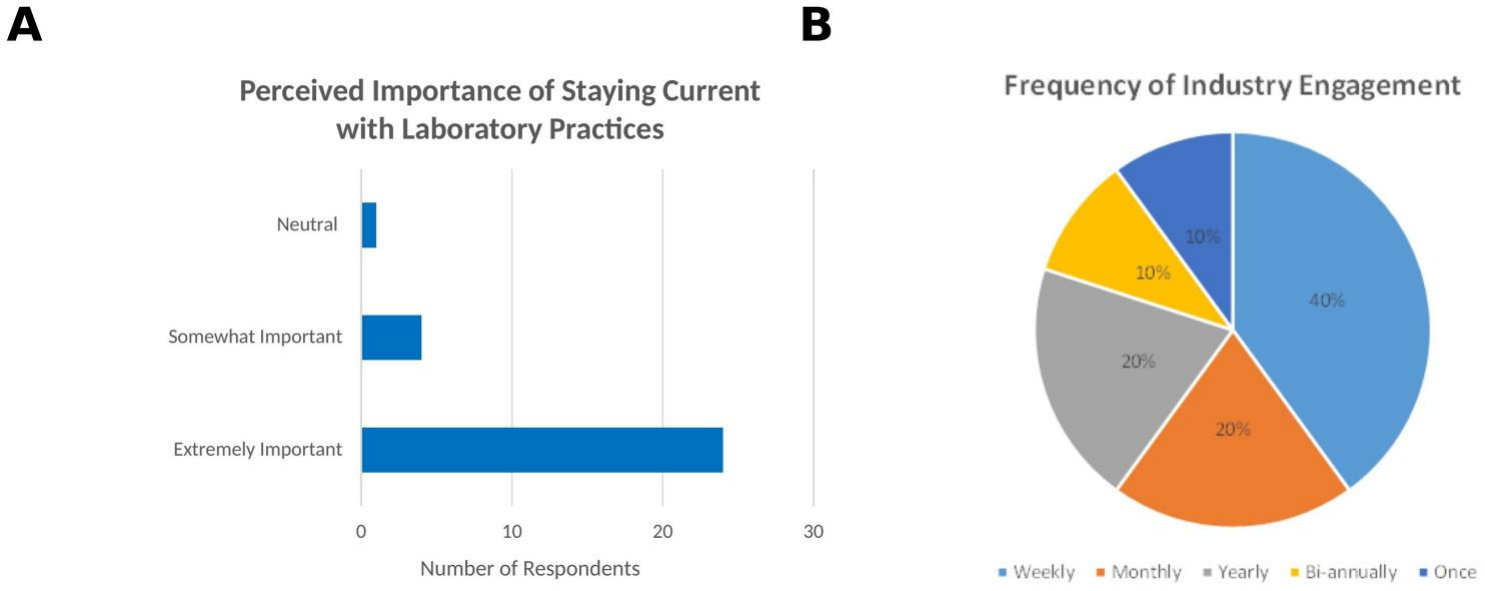
Importance of clinical currency and current engagement with industry among Medical Laboratory Science academics (*n* = 29). (**A**) Distribution of respondents’ ratings of the importance of staying up-to-date with current laboratory practices. (**B**) Current frequency of respondents’ engagement with medical laboratories or industry professionals.

Medical Laboratory Science academics expressed a strong commitment to maintaining up-to-date industry knowledge and remained engaged with clinical practice. As presented in **Figure 1A**, the majority of respondents (20/29 [69%]) rated staying current with laboratory practice as “extremely important.” Six (21%) respondents rated it as “somewhat important,” and 3 (10%) as “neutral.” In terms of current engagement with industry (**Figure 1B**), most academics (18/29 [62%]) reported contact with medical laboratory professionals weekly, 7 (24%) described monthly engagement, 3 (10%) indicated contact every 6 months to a year, and only 1 respondent (3%) reported no engagement. These findings reinforce the prevailing view among MLS academics of the centrality of ongoing industry involvement to their professional practice.

### Perceptions of academic work placement: benefits, impact, and interest

#### Perceived benefit for teaching

**Figure 2:**
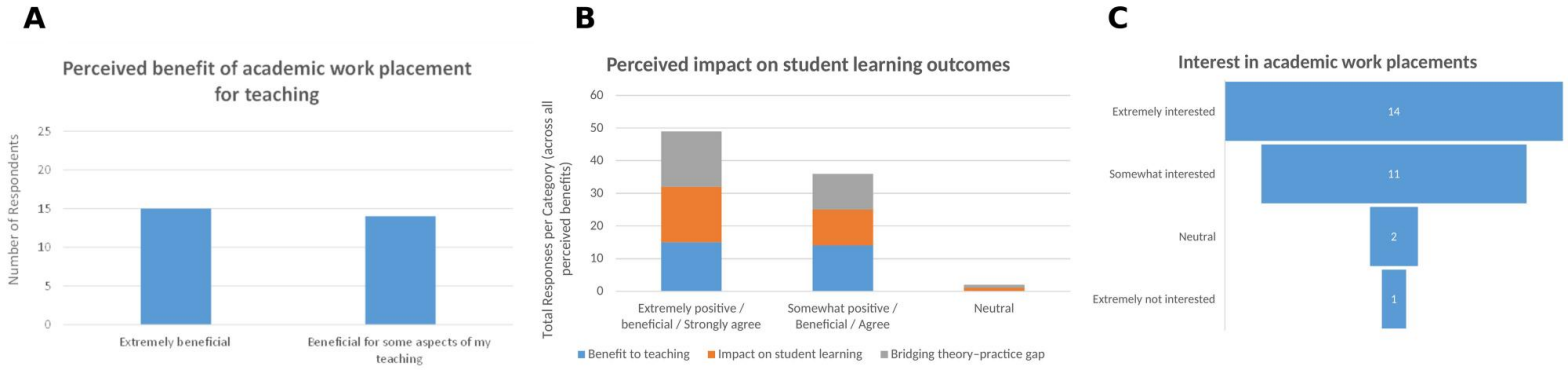
Perceived educational value of academic clinical placements and interest in participation among Medical Laboratory Science academics (*n* = 29). (**A**) Perceived benefit of a work placement in a medical laboratory for teaching. (**B**) Perceived impact of academic clinical placements on student learning outcomes. (**C**) Respondents’ interest in participating in a clinical placement to enhance teaching.

Respondents reported considerable perceived benefits of academic work placements for their teaching practice (**Figure 2A**). Half the academics (15/29 [52%]) described such placements as “extremely beneficial” for their teaching, while the remaining 14 (48%) noted that placements were “beneficial for some aspects.” No respondent reported placements as not beneficial, suggesting strong positive consensus.

#### Perceived impact on student learning outcomes

A similarly positive trend was seen regarding perceived impacts on student learning outcomes (**Figure 2B**). Seventeen (59%) academics described the impact as “extremely positive,” 11 (38%) as “somewhat positive,” and only 1 (3%) considered the impact to be “neutral.” None felt that placements negatively affected student outcomes.

#### Interest in participation (MLS academics)

Interest in participating in an academic work placement as a faculty professional development activity was high (**Figure 2C**). Fifteen (52%) respondents indicated that they were “extremely interested” in undertaking such placement themselves, 10 (34%) were “somewhat interested,” and 4 (14%) were either “neutral” or “not interested.” This strong interest signals a readiness among academics to engage in further workplace-based professional development.

### Program leaders’ perceptions of academic clinical placements

**Figure 3:**
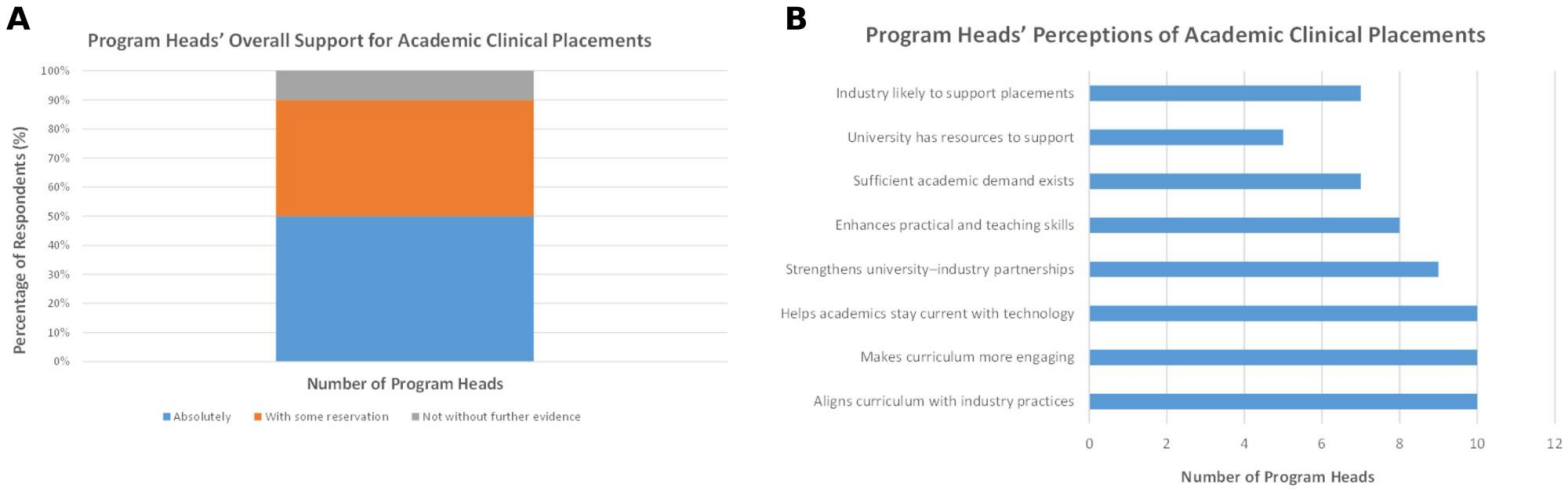
Program leader perspectives on academic clinical placements (*n* = 10). (**A**) Overall level of support for implementing clinical placements for Medical Laboratory Science academics (absolutely supportive, supportive with reservations, or not supportive without further evidence). (**B**) Program leader agreement with statements regarding potential benefits and feasibility, including curriculum alignment with industry practice, student engagement, technological currency, university-industry partnerships, skill development, perceived academic demand, university resourcing, and perceived industry support.

Program leaders were similarly supportive of academic clinical placements. As summarized in **Figure 3A**, half (5/10 [50%]) were absolutely supportive, with some reservations, and 1 (10%) respondent indicated a need for further evidence before providing support. **Figure 3B** shows that all program leaders (10/10) agreed on the educational value of placements, specifically highlighting benefits such as enhanced curriculum alignment with industry practice, increased student engagement, and improved maintenance of technological currency among academics. There was also broad consensus that placements have the potential to strengthen university-industry partnerships and support academics’ professional skill development. Despite these positive perceptions, views of feasibility were mixed: 70% of program leaders agreed that there was sufficient academic demand and anticipated industry support, but only 50% felt their university had the resources to support implementation, pointing to potential institutional barriers.

### Departmental support and likelihood of participation in academic placement

**Figure 4:**
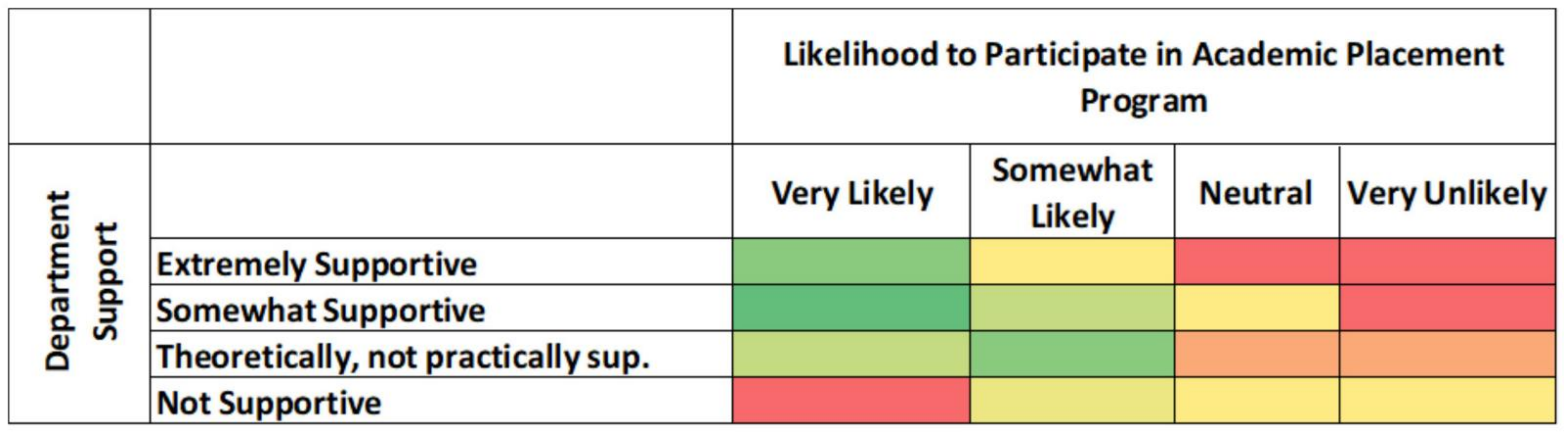
Relationship between departmental support and likelihood of participation in academic placement programs. Heatmap showing distribution of respondents by department support level (rows) and likelihood to participate (columns) in academic placement programs. Cell color corresponds to response frequency, with green indicating the most common combinations, yellow representing moderate frequency, and red the least common. The highest concentration of responses appears where support is rated as “supportive” and likelihood of participation is greatest.

**Figure 5:**
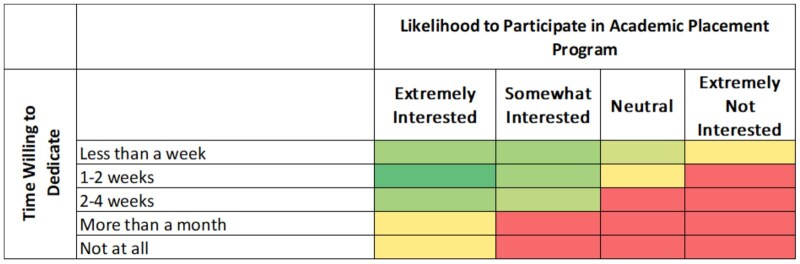
Likelihood of participation in academic placement by time willing to dedicate. Heatmap depicting the frequency of responses across combinations of likelihood to participate (rows) and time willing to dedicate (columns) to an academic placement program. Green indicates the highest frequencies, yellow shows moderate responses, and red denotes the least common. Most frequent responses are associated with high interest and shorter time commitments.

**Figures 4 and 5** further explore factors influencing academics’ likelihood to participate in placement programs. As shown in **Figure 4**, higher likelihood of participation was associated with greater perceived departmental support; respondents who rated their department as “supportive” were more likely to consider participating than those who reported less tangible support. **Figure 5** illustrates that the intention to participate was highest for shorter placements, with most respondents “very likely” or “somewhat likely” to participate if the placement lasted 2 weeks or less, while likelihood diminished as the proposed duration increased. These results suggest that both organizational and manageable placement durations are critical factors for encouraging academic uptake of clinical placement opportunities.

## Discussion

This study offers new insight into the perceived value and feasibility of industry clinical placements for MLS academics by integrating the perspectives both of academics and of program leaders across AIMS-accredited programs in Australia. The findings reveal strong alignment between these groups regarding the educational benefits of academic clinical placements, especially in enhancing curriculum relevance, maintaining technological currency, and strengthening university-industry partnerships. Notably, although enthusiasm and interest in participation were high among academics and leaders, concerns regarding institutional resources and implementation capacity were prominent, suggesting the practical barriers that may limit uptake despite positive perceptions.

The MLS academics overwhelmingly reported that industry clinical placements were beneficial for their professional development and teaching effectiveness. Most respondents perceived positive impacts on student learning, highlighting the value of placements in bridging theoretical knowledge and clinical practice. These outcomes are consistent with broader health professions education literature, which underscores the importance of authentic industry engagement for maintaining discipline currency and advancing pedagogical quality.^14^^,^^19^ Although continuing professional development activities may support professional currency, the academic placements considered in this study were framed as short periods of direct workplace immersion intended to provide exposure to contemporary laboratory workflows, technologies, and quality systems, complementing other forms of professional development. In particular, the literature increasingly advocates for academic professional development activities that move beyond traditional scholarship to include practice-based learning and engagement with contemporary workplace environments.^20^^,^^21^ This strong interest in short, periodic placements further underscores a desire for flexible professional development opportunities that can be accommodated within existing academic workload structures.

Support from program leaders was similarly robust, with unanimous agreement that academic clinical placements would improve curriculum alignment with current industry practices and enhance technological currency. This leadership endorsement is important given leaders’ roles in curriculum governance, accreditation, and resource allocation. Only half the program leaders, however, felt that their universities had adequate resources to support academic placements. This divergence between perceived educational value and practical feasibility reflects a well-documented challenge in academic-industry engagement, where enthusiasm often exists alongside substantial institutional and logistical barriers.

Differences in perceived teaching impact between academics and program leaders also emerged. Although most program leaders acknowledged that placements could improve practical and teaching skills, some expressed reservations about direct enhancements to teaching effectiveness, contrasting with academics’ strong belief in these benefits. This discrepancy may reflect different assumptions about how professional development influences pedagogical practice or may indicate the absence of formal mechanisms for evaluating the specific teaching outcomes resulting from academic-industry engagement.^22^ The literature suggests that structured placement models linking clinical experiences explicitly to teaching objectives, reflective practice, and curriculum development may help demonstrate and maximize pedagogical impact.^10^^,^^22^

Both groups favored short, flexible placements (usually 1 to 2 weeks annually or biannually), indicating a preference for models that balance professional development with existing academic demands.^12^^,^^13^ Integrating these placements into institutional professional development frameworks and recognizing them formally within academic workloads could support sustainability and uptake. Moreover, establishing such flexible and well-supported placement models not only meets the needs of academics but also facilitates stronger university-industry partnerships. These partnerships are increasingly critical for sustaining high-quality MLS education, particularly in the context of rapid technological change and evolving workforce requirements.^7^^,^^11^^,^^12^ Academic placements offer reciprocal benefits; they support industry involvement in curriculum design, foster academics’ awareness of contemporary practice environments, and encourage collaborative education innovation.^18^

### Implications for practice

Our findings suggest that structured, short-duration clinical placements are both desirable and potentially achievable as a component of academic professional development in MLS. Institutional support, workload recognition, and formalized partnerships with industry laboratories will be vital for successful implementation. Establishing pilot programs and evaluating their outcomes could help address leadership concerns and facilitate broader adoption. A key implementation consideration is ensuring that faculty placements do not affect existing student placement capacity. Feasible approaches may include scheduling academic placements outside peak student placement periods, using short observational or shadowing models with clearly defined objectives, and partnering with sites that are not currently hosting student cohorts.

### Limitations

Despite its strengths, this study has several limitations. The modest sample size (29 academics and 10 program leaders) and reliance on self-reported perceptions may limit generalizability and introduce response bias because individuals with stronger views or greater engagement may have been more likely to participate. Future research should include larger, multi-institution samples and use longitudinal or mixed-method designs to evaluate the impact of academic clinical placements on teaching practice, curriculum development, and student outcomes.

## Conclusion

Industry clinical placements are widely perceived by both MLS academics and program leaders as educationally valuable, enhancing curriculum relevance, professional development, and student learning. Practical considerations, however, particularly with respect to resourcing and institutional support, remain notable barriers. Addressing these challenges through structured placement models and dedicated institutional backing may enhance the sustainability and impact of academic clinical placements in MLS education.

## Data Availability

The data underlying this article will be shared on reasonable request to the corresponding author.
